# Response of Soil Fungal Community to Reforestation on Shifting Sand Dune in the Horqin Sandy Land, Northeast China

**DOI:** 10.3390/microorganisms12081545

**Published:** 2024-07-28

**Authors:** Chengyou Cao, Ying Zhang, Zhenbo Cui

**Affiliations:** 1 College of Life and Health Sciences, Northeastern University, Shenyang 110169, China; zhangying@mail.neu.edu.cn (Y.Z.); cuizhenbo@mail.neu.edu.cn (Z.C.); 2 Liaoning Province Key Laboratory of Bioresource Research and Development, Northeastern University, Shenyang 110169, China

**Keywords:** soil fungal diversity, sand-fixation plantation, microbial community succession, desertification control

## Abstract

Reforestation of native shrub on shifting sand dunes has been widely used for desertification control in semi-arid grassland in Northeast China. Previous studies have confirmed that plantation establishment facilitates fixing sand dunes, restoring vegetation, and improving soil properties, but very few have focused on the response of the soil fungal community. In this study, a chronosequence of *Caragana microphylla* (CM) shrub sand-fixation plantations (8-, 19-, and 33-year-old), non-vegetated shifting sand dunes (0 years), and adjacent natural CM forests (NCFs; 50-year-old) in the Horqin sandy land were selected as experimental sites. Soil properties including enzymatic activities were determined, and the composition and structure of the soil fungal community were investigated using the Illumina MiSeq sequencing technique based on the internal transcribed spacer (*ITS*) rDNA. This study aimed to (1) describe the response of the soil fungal community to revegetation onto a moving sand dune by planting a native shrub plantation; (2) determine the main soil factors driving the succession of the fungal community; and (3) discuss whether the soil fungal community can be restored to its original state by reforestation. The reforestation of CM significantly ameliorated soil properties, increased soil fungal diversity, and altered the composition and structure of the soil fungal community. Ascomycota, Basidiomycota, and Zoopagomycota were the dominant phyla in all sites. Ascomycota did not respond to plantation development, whereas the other two dominant phyla linearly increased or decreased with the plantation age. The relative abundance of dominant genera varied with sites and showed a waning and waxing characteristic. The composition and structure of the soil fungal community in the 33-year CM plantation were very close to that of the NCF, indicating the restorability of the soil fungal community. The succession of the soil fungal community was directly driven by soil properties, of which soil moisture, organic matter, total N, urease, and protease were the main affecting factors.

## 1. Introduction

Fungi are a crucial functional component of the soil system and play important roles in carbon turnover, the acquisition and cycling of N and P, belowground carbon sequestration, and soil development [1,2]. Soil fungi also indirectly affect ecosystem function by forming mutual symbiosis with plants, pathogenically attacking some plant species, and altering plant–plant interspecific competition and the feedback of plant–soil interactions [3,4]. Therefore, the soil fungal community can significantly affect the restoration of a degraded ecosystem and can be used as an indicator for the assessment of ecological restoration, but it is often overlooked in practice [5,6]. The functions of fungal communities in the restoration of terrestrial ecosystems mainly depend on their diversity and composition structure, e.g., more mycorrhizal fungi facilitate increases in the availabilities of soil N and P, which aids in of plant and microbial growth [4,7]. Some studies reported that adding fungal inocula in degraded lands can increase productivity and alter plant community composition [8,9,10], indicating the importance of soil fungi in restoration practice. Understanding the diversity and composition of the soil fungal community and the main soil factors that affect community succession can help to evaluate the ecological benefits of degraded land restoration and facilitate the conservation of fungal diversity in fragile ecosystems. Despite increasing soil microbial diversity studies over the past decades, the knowledge of most belowground microorganisms remains limited because of their microscopic size, hidden existence, and the lack of accurate detecting methods. Traditional culture-based methods cannot obtain the most accurate compositions of the soil fungal community. Therefore, soil fungal diversity is still underestimated [11]. Molecular techniques can offer great opportunities to accurately describe the diversity and composition of the soil fungal community and enhance our understanding of belowground biodiversity [12].

Desertification, a global environmental issue, has been extensively studied all around the world because of its wide distribution, serious damage to the environment, and the great economic loss it produces [13]. Revegetation on desertified land has been confirmed to be an effective strategy for desertification control [14,15,16] and has been adopted in many countries. The Horqin sandy land (also called Horqin grassland), covering an area of 5.18 × 10^4^ km^2^, is located in the semi-arid agropastoral transitional zone of Northeast China and was historically suitable for animal husbandry industries. However, it has suffered severe desertification since the 1970s, primarily because of overgrazing, excessive reclamation, and the overcollection of firewood induced by the pressure of local resident increases [14]. To control land desertification, planting drought- and sand-enduring trees or shrubs on moving sandy land with the help of high-density straw checkerboard barriers has been widely used in this region. With the development of a sand-fixation plantation, moving sand land was fixed, some understory herbaceous plants subsequently invaded, and the structure and function of the vegetation were gradually restored. Revegetation onto moving sand dunes can result in the variations in soil properties, nutrient content, and microbial communities. Many studies have confirmed that the establishment of sand-fixation plantations alters the microclimate, increases soil nutrients including organic matter, total and available nitrogen (N), phosphorus (P), and potassium (K), improves soil biological activity, and ameliorates soil physicochemical properties [17,18,19]. Soil microbial communities are highly sensitive to environmental variations. During sand-fixation plantation development, the changing soil condition would result in an unpredictable microbial community structure due to the different responses of dominant taxa to the niche change. Plantation types, characteristics of habitats, and the quality of plant inputs may significantly influence soil microbial communities during revegetation on moving sandy dunes. Yang et al. [20,21] reported that soil organic carbon and plant species are the key factors regulating soil fungal diversity in China’s Loess Plateau and the grassland of the Tibetan plateau. Meanwhile, soil nutrients including organic matter, total and available N, and available phosphorus also significantly affect fungal diversity [22,23,24]. Recently, several studies have employed molecular biological methods and high-throughput sequencing techniques to detect the responses of soil bacterial communities involving N cycle and P turnover to the revegetation on moving sand land [25,26], but very few have focused on the soil fungal community. The response of the soil fungal community to the establishment of the sand-fixation plantation is still unknown. Information on the dominant composition of the soil fungal community and its varying tendencies is required for a better understanding of vegetation restoration mechanisms and plant–soil interactions. In this study, we selected a chronosequence of a *Caragana microphylla* shrub sand-fixation plantation in the Horqin sandy land to investigate the composition and structure of the soil fungal community using the Illumina MiSeq sequencing technique based on the internal transcribed spacer (*ITS*) rDNA. The objectives of the study were to (1) describe the soil fungal community response to revegetation onto a moving sand dune by planting a native shrub plantation; (2) determine the main soil factors driving the succession of the fungal community; and (3) discuss whether the soil fungal community can be restored to its native state by reforestation. We hypothesized that the fungal diversity increased with plantation age, and the dominant taxa significantly varied with the plantation development and soil property improvement.

## 2. Materials and Methods

### 2.1. Study Location and Site Description

This study was conducted at the Wulanaodu Station of Desertification Control (43°02′ N, 119°39′ E) of the Chinese Academy of Sciences, western Horqin sandy land. The Wulanaodu region has a semi-arid continental monsoon climate. The average annual precipitation, temperature, and frost-free period are 340.5 mm, 6.3 °C, and 130 d, respectively. Windy-sandy weather frequently occurs in winter and spring, and the annual average wind velocity is 4.5 m s^−1^. The original vegetation was elm steppe-woodland; however, most grasslands have been desertified in recent decades, which has resulted in the destruction of the original vegetation. At present, the landscape is characterized as a mosaic of desertified grassland, fixed sand dunes, shifting sand dunes, and interdune lowlands [26]. The dominant native plants include *Artemisia halodendron*, *C. microphylla*, *Salix gordejevii*, *Hedysarum fruticosum*, *Astragalus adsuregens*, *Potentilla filipendula*, *Lespedeza davurica*, *Pennisetum flaecidum*, and *Setaria viridis.* The soils were classified as Cambic Arenosols [27]. To control desertification, some native shrub plantations (e.g., *S. gordejevii*, *C. microphylla*, and *H. fruticosum*) were commonly planted on mobile sand dunes under the protection of a straw checkerboard barrier (1 m × 1 m squares). Up to now, a large area of the sand-fixation plantation has been gradually established around the Wulanaodu region.

### 2.2. Soil Sampling

The soils were sampled in August 2022. Soil samples from an age sequence of a *C. microphylla* plantation (8-, 19-, and 33-year-old), adjacent non-vegetated shifting sand dunes, and natural *C. microphylla* forests (designated as CM8, CM19, CM33, SSD, and NCF, respectively) were selected as experimental sites. The SSD was formed from long-term wind erosion and eolian deposition of sandy grassland and can be considered as the original state before plantation establishment (as the control). The NCF was natural grassland with some sparsely distributed CM clumps, and it has been fenced in by barbed wire for 50 years. Morphological traits of different sites are shown in Table 1. Three sites of the plantation of each age, SSD, and NCF were set up for sampling. For each site, one 30 m × 30 m plot was established, and five representative CM clumps were selected and sampled. Each plot was 300 m away from the other one. Under the crown of each clump, subsamples (0–10 cm) were collected from four directions, and then a total of 20 subsamples in the plot were pooled as a sample. Half of each sample was air-dried for the analysis of physical and chemical soil properties, and the other half was frozen at −80 °C in a refrigerator for the analysis of biological activity and DNA extraction.

### 2.3. Soil Property Determination

Soil moisture (SM) was gravimetrically determined by drying the soil at 105 °C for 24 h. The air-dried soil was ground for the analyses of pH (soil: water ratio 1:2.5), electrical conductivity (soil: water ratio 1:5; EC), soil organic matter (SOM), total nitrogen, phosphorus, and potassium (TN, TP, and TK), NH_4_-N, and available phosphorus (AP) and potassium (AK). SOM and TN were determined using the K_2_Cr_2_O_7_–H_2_SO_4_ oxidation and the semimicro-Kjeldahl digestion methods, respectively. Soil TP and AP were measured using the acid digestion molybdate colorimetric method and the molybdate ascorbic acid method (in 0.5 M NaHCO_3_), respectively. TK and AK were measured using an atomic absorption spectroscopy method. NH_4_-N was extracted using 1 M KCl solution and determined using an automated discrete analyzer (CleverChem 380, Hamburg, Germany). The measurement procedures of the above soil factors were all according to methods described in Lin [28].

Soil urease activity was determined using the method of Kandeler and Gerber [29]. Protease activity was determined according to the method of Ladd and Bulter [30]. Glucosidase activity was determined using the method described by Xu and Zheng [31]. The activity of soil alkaline phosphomonoesterase (APA) was measured using the original method of Tabatabai [32], with some modifications by Sardans and Peñuelas [33]. The activities of polyphenol oxidase (POA) and dehydrogenase were measured following the method described by Perucci et al. [34] and ISSCAS [35], respectively. The detailed procedure is described in the Supplementary Materials.

### 2.4. DNA Extraction, ITS rDNA Sequencing, and Data Processing and Analysis

Microbial DNA was extracted from 0.3 g fresh soil sample using a soil DNA extraction kit (Sangon Biotech, Shanghai, China) following the manufacturer’s instructions. Next-generation sequencing library preparations and Illumina MiSeq sequencing were conducted at GENEWIZ, Inc. (Suzhou, China). The QIIME (Quantitative Insights Into Microbial Ecology) data analysis package (http://qiime.org/, accessed on 20 January 2023) was used for *ITS* rDNA data analysis. Sequences were grouped into operational taxonomic units (OTUs) using the clustering program VSEARCH (1.9.6) against the UNITE *ITS* database (https://unite.ut.ee/, accessed on 20 January 2023) at a 97% cutoff. The classification of fungi was according to the taxonomy system of Kirk et al. [36]. Sequences were rarefied prior to calculation of alpha and beta diversity statistics. Alpha diversity indexes including the Shannon–Wiener index (SW), Chao’s species richness estimator (Chao), and abundance-based coverage estimator (ACE) were calculated in QIIME2 from rarefied samples. Hierarchical clustering analysis was performed, and an unweighted pair group method with arithmetic means (UPGMA) tree was built to differentiate the fungal community structures of different sites. Raw sequencing datasets were deposited in the NCBI Sequence Read Archive (SRA) under the accession number PRJNA859000. More details of the sequencing procedure and data analysis are provided in the Supplementary Materials.

### 2.5. Regression Analysis and Redundancy Analysis (RDA)

The soil properties of different sites were analyzed by one-way ANOVA and multiple comparisons followed by Fisher’s LSD test. The responses of soil properties, dominant fungal taxa, and alpha diversity of the fungal community to plantation development were fitted by a linear regression model using the SPSS (version 18.0) software package. *p* < 0.05 was considered statistically significant. RDA was performed using CANOCO 5.0 for Windows (Biometris-Plant Research International, Wageningen, The Netherlands) to determine which soil factor significantly affected fungal communities, and the correlations of the soil factors were examined by a Monte Carlo permutation.

## 3. Results

### 3.1. Improvement of CM Plantation on Soil Properties

The values of soil pH, SM, EC, SOM, TN, TP, TK, NH_4_-N, AP, and AK of different sites are shown in Table 2. These soil factors all significantly linearly increased with the plantation age (*p* < 0.05). Their values in CM8, CM19, and CM33 sites were 1.01–1.03, 1.80–4.23, 1.65–2.27, 4.40–12.53, 5.89–7.67, 1.50–2.25, 1.11–1.18, 1.37–1.89, 1.20–1.49, and 1.05–1.11 times higher than those in SSD site, respectively, which indicated that planting the CM plantation on the SSD can significantly ameliorate soil properties and facilitate increases in soil nutrients, especially SOM and TN. The activities of soil urease, protease, alkaline phosphomonoesterase (APA), glucosidase, polyphenol oxidase (POA), and dehydrogenase showed similar varying tendencies, and significantly linear relationships between enzymatic activities and plantation age were also observed (*p* < 0.01, Table 2). The activities of selected soil enzymes under CM plantations were 5.95–30.41, 2.50–12.01, 7.59–37.22, 3.07–6.19, 1.41–2.37, and 1.91–5.23 times higher than those in the SSD, respectively (Table 2). No significant differences in SM, pH, TK, NH_4_-N, AK, glucosidase, dehydrogenase, or POA were observed between CM33 and NCF. However, EC, SOM, TN, TP, AP, urease, APA, and protease were significantly higher in NCF than those in CM33.

### 3.2. Variation in Soil Fungal Diversity along Plantation Development

**Figure 1:**
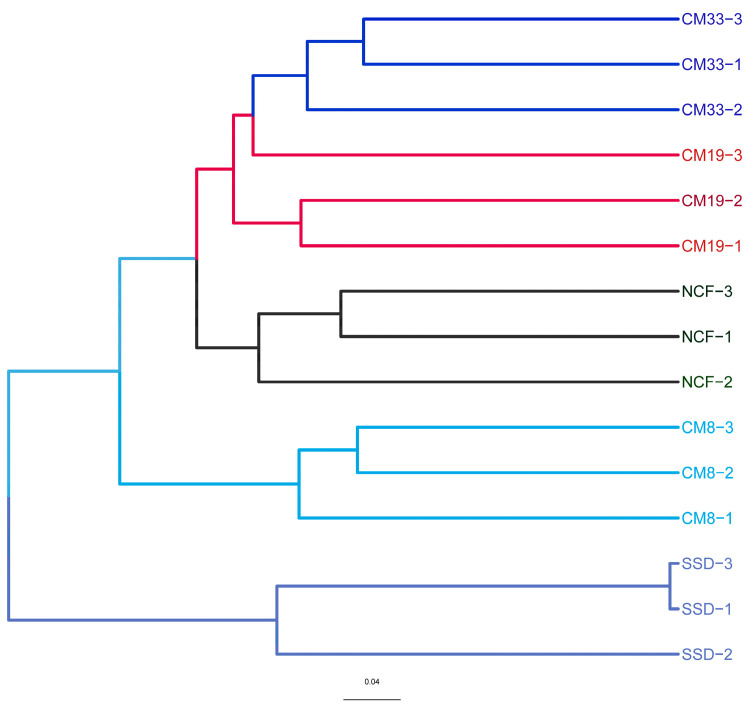
Cluster analysis based on UPGMA of the structures of soil fungal communities under different sites. SSD: shifting sand dune (control); CM8, CM19, and CM33: 8-, 19-, and 33-year *C. microphylla* plantation, respectively; NCF: natural *C. microphylla* forest.

A total of 1,476,350 high-quality *ITS* gene sequences were obtained from 15 samples through MiSeq sequencing analysis. All rarefaction curves tended to approach a plateau at the sequencing depth (Figure S1), suggesting that the sequencing depth was enough to detect the fungal community composition. The alpha diversity indexes (including SW, Chao, and ACE) were calculated based on rarefied samples. All diversity indexes were significantly higher in vegetation-covered sites than the SSD and showed linearly increasing tendencies with plantation age (*p* < 0.05). The average observed OTUs in the SSD, CM8, CM19, CM33, and NSF were 63.7, 388.8, 426.5, 508.6, and 556.5, respectively, also showing a linearly increasing trend (Table 3). No significant differences in all indexes between CM33 and NSF were found. Hierarchical clustering analysis divided all samples into five groups. The three samples from the same site were individually clustered in a group, and the samples from different sites were grouped into another one, showing the differences in the structures of soil fungal communities along plantation development (Figure 1).

### 3.3. Taxonomic Compositions of Soil Fungal Communities across Plantation Development

**Figure 2:**
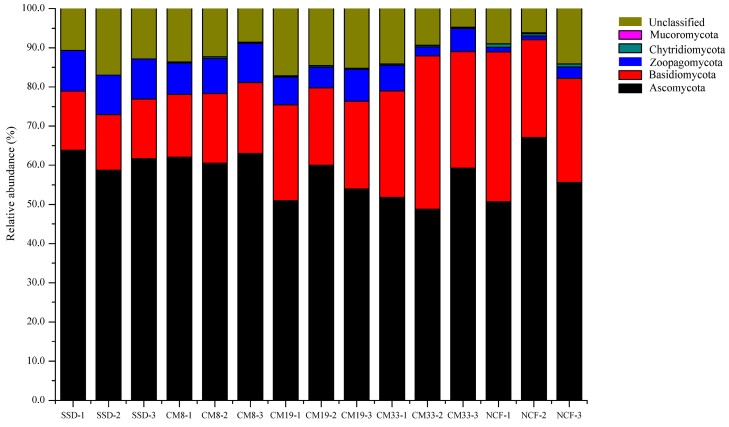
The relative abundance of soil fungal phyla in different sites. The results of ANOVA of the relative abundance for each phylum in response to age: Ascomycota: *R*^2^ = 0.136, *F*_regression_ = 2.054, *p* = 0.175; Basidiomycota: *R*^2^ = 0.655, *F*_regression_ = 24.456, *p* < 0.001; Zoopagomycota: *R*^2^ = 0.874, *F*_regression_ = 90.466, *p* < 0.001; Chytridiomycota: *R*^2^ =0.789, *F*_regression_ = 48.579, *p* < 0.001; Mucoromycota: *R*^2^ = 0.151, *F*_regression_ = 2.313, *p* = 0.152. SSD: shifting sand dune (control); CM8, CM19, and CM33: 8-, 19-, and 33-year *C. microphylla* plantation, respectively; NCF: natural *C. microphylla* forest.

All obtained fungal OTUs can be classified into five different phyla, 41 orders, 62 families, 104 genera, or 148 species. The phyla of Ascomycota, Basidiomycota, and Zoopagomycota were detected in all samples, with the total relative abundance accounting for 82.56% to 91.18%. Ascomycota was most dominant phylum in all communities, with the relative abundance ranging from 53.26% to 61.89%, and no significant difference was found among the sites (Figure 2). The relative abundance of Basidiomycota and Zoopagomycota ranged from 14.83% to 32.04% and from 1.71% to 10.29%, respectively, and both significantly linearly increased with the plantation age (*R*^2^ = 0.655 and 0.874, respectively, *p* < 0.001). Chytridiomycota was also observed in vegetation-covered sites. Although its relative abundance was <1% in all samples, a tendency of linear increase with plantation age was also found (*R*^2^ = 0.789, *p* < 0.001). Mucoromycota was only detected in CM19, CM33, and NCF, with a relative abundance < 0.1%.

**Figure 3:**
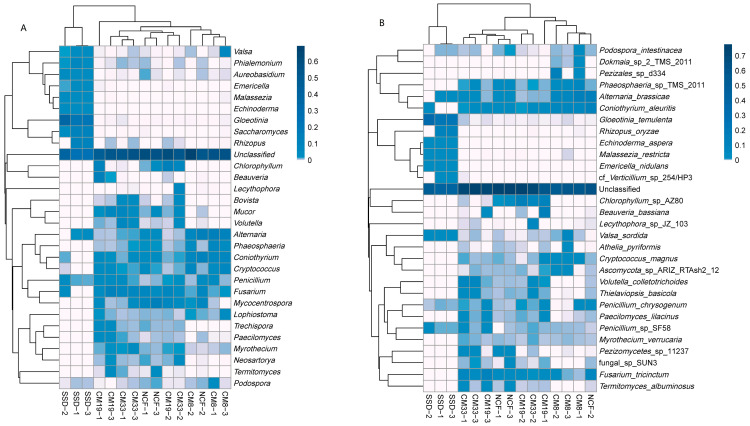
Fungal distribution of top 30 most abundant genera (**A**) or species (**B**) of different samples. The heatmap shows the relative abundance of fungal genera or species (variables clustering on the vertical axis); and the relative values for genera or species are expressed by color intensity. SSD: shifting sand dune (control); CM8, CM19, and CM33: 8-, 19-, and 33-year *C. microphylla* plantation, respectively; NCF: natural *C. microphylla* forest.

The numbers of detected genera in SSD, CM8, CM19, CM33, and NCF were 22, 71, 83, 98, and 95, respectively, showing an increasing trend with sand-fixation plantation development. Based on relative abundance, 30 dominant genera were selected to draw a heat map (Figure 3A), which shows the frequently observed fungal genera in different sites. The sum of the relative abundance of the 30 dominant genera accounted for 35.75% in CM8 to 67.03% in the SSD site. The dominant genera were different in different sites. In the SSD, the dominant genera included *Gloeotinia* (relative abundance 25.10%), *Saccharomyces* (8.31%), *Rhizopus* (6.90%), *Phialemonium* (4.11%), *Malassezia* (4.02%), and *Echinoderma* (3.76%). However their relative abundance all significantly decreased in vegetation-covered sites (<0.1%): *Alternaria* (9.4%), *Coniothyrium* (6.8%), *Cryptococcus* (6.04%), and *Phaeosphaeria* (4.29%) were the dominant genera in CM8; *Chlorophyllum* (7.66%), *Fusarium* (5.84%), and *Beauveria* (5.00%) were the dominant genera in CM19; *Fusarium* (5.89%), *Mucor* (4.08%), and *Volutella* (4.51%) were the dominant genera in CM33 site; and *Alternaria* (5.72%), *Coniothyrium* (4.67%), and *Mycocentrospora* (8.81%) were the dominant genera in NSF sites. This phenomenon indicated the waning and waxing characteristic of dominant fungal taxa during the sand-fixation plantation development. According to Figure 3A, CM19, CM33, and NCF exhibited similar distribution patterns of higher-abundance genera.

Although most OTUs could not be classified at the species level in the database, 21, 110, 113, 130, and 139 fungal species were detected in the SSD: CM8, CM19, CM33, and NCF, respectively. *Gloeotinia temulenta*, *Rhizopus oryzae*, *Malassezia restricta*, and *Echinoderma aspera* were the dominant fungal species in the SSD; and in the CM and NCF sites, *Alternaria brassicae*, *Coniothyrium aleuritis*, *Fusarium tricinctum*, *Phaeosphaeria* sp. TMS_2011, *Beauveria bassiana*, *Volutella colletotrichoides*, and *Chlorophyllum* sp. AZ80 were the dominant species. A heat map was drawn based on the relative abundance of the top 30 dominant species in different samples (Figure 3B).

### 3.4. Dependence of Fungal Community Structure on Soil Property

**Figure 4:**
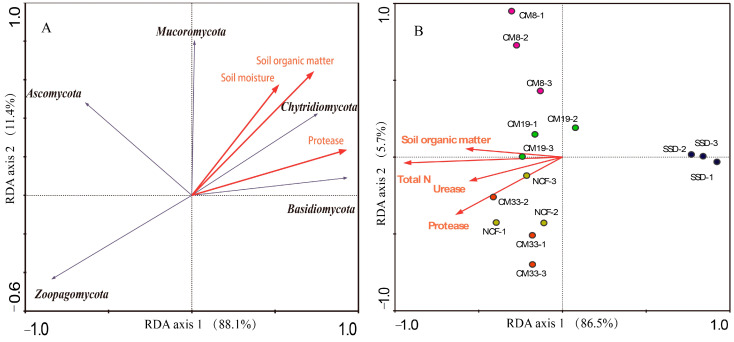
Redundancy analysis (RDA) between soil fungal community structure and soil properties: (**A**) phylum composition; (**B**) dominant generic composition. The correlations of the soil parameters were examined by a Monte Carlo permutation. SSD: shifting sand dune; CM8, CM19, and CM33: 8-, 19-, and 33-year *C. microphylla* plantation, respectively; NCF: natural *C. microphylla* forest.

RDA was performed to assess the relationship between the soil fungal community structure (the relative abundance of phylum and top 30 dominant genera) and soil properties (SM, pH, EC, SOM, TN, TP, TK, NH_4_-N, AP, AK, urease, protease, APA, glucosidase, POA, and dehydrogenase). The result showed that 88.1% and 11.4% of the variations in compositions of fungal phylum can be explained in the first and second axis, respectively; and those in dominant genus composition were explained by 86.5% and 5.7%, respectively (Figure 4). Figure 4 also shows that the total samples can be clustered in three groups (SSD, CM8 and CM19, and CM33 and NCF), indicating the gradual variations in the structures of soil fungal communities along the plantation development. Soil moisture, organic matter, and protease were selected by the Monte Carlo permutation test of RDA, which significantly affected the phylum composition of soil fungi, especially Chtridiomycota and Basidiomycota (Figure 4A). Soil organic matter, TN, urease, and protease were considered the main factors affecting fungal generic composition (Figure 4B; *p* < 0.05).

## 4. Discussion

### 4.1. Amelioration of Soil Properties and Microbiological Properties via Reforestation on Shifting Sand Dunes

Seriously desertified soil is loose in consistency, poor in nutrients, low in water-holding capacity, and unstable in topsoil, which is apt to be wind-eroded and is detrimental to the survival of native plants and soil microorganisms. Reforestation by sowing native shrubs on shifting sand dunes with the help of a high-density sand-protecting barrier has been considered a successful method for vegetation restoration [14]. This study indicated that the revegetation of the CM shrub plantation significantly improved the physical, chemical, and microbiological properties of the soil, and the improvement effect increased with the plantation development. This phenomenon is a continuous and complicated process that was simultaneously driven by many biotic and abiotic factors. The increase in soil nutrients mainly depended on following: (1) the alterations of the microenvironment, including the balance of near-surface heat and water, wind velocity, ground roughness, and surface albedo [17], which facilitated the accumulation and transformation of soil organic matter; (2) the increasing input of litter from CM and many annual and perennial herbs that gradually invaded and increased under the canopy [18]; (3) the interception of fine soil particles and atmospheric dust fall that are rich in nutrients by plantations; (4) the increase in the quantity of soil microorganisms, especially some functional microbial taxa involved in the N-cycle, organic phosphate mineralization, and inorganic phosphate dissolution [25,26], which accelerated the rates of SOM mineralization and the decomposition of litter and dead roots, thereby increasing the bioavailability of soil N, P, and K.

Soil enzymes, mainly produced by microbial metabolism and plant root secretion, catalyze various soil redox and hydrolysis reactions of different complicated compounds. They play important roles in nutrient cycling processes, including the decomposition of litter and the form transformation of nutrient elements, and are therefore commonly used as an indicator for evaluating microbial function and soil quality [37]. Recovery of vegetation and the soil microbial community in sandy soil facilitated the increases in the activities of various soil enzymes. The increases in soil nutrients, especially SOM and TN, are favorable for the survival and reproduction of fungi, which can result in increases in fungal diversity and enzymatic activities and thereby enhance the rates of nutrients cycles. Yang et al. [38] reported that soil urease activity was positively correlated with fungal diversity. Soil urease can accelerate the generating rate of available N, which can be directly assimilated by microbes and plants. In this study, the activities of assayed soil enzymes including urease, protease, alkaline phosphomonoesterase, glucosidase, polyphenol oxidase, and dehydrogenase all linearly increased along CM plantation development (Table 2). Increased soil enzymatic activities can improve oxidation-reduction conditions and accelerate the mineralization rates of soil organic C, N, and P compounds. Meanwhile, the varying tendency of soil enzymatic activity was similar to that of soil nutrients and fugal diversity. Several studies reported that soil alkaline phosphomonoesterase activity was significantly associated with *phoD*-harboring microbial quantity in a chronosequence of the sand-fixation plantation [26]. Therefore, soil enzymatic activity is closely correlated with soil nutrients, the quantities of functional microbial groups, and the structure of the microbial community.

### 4.2. Recovery of Soil Fungal Community during Reforestation on Shifting Sand Dunes

The composition and structure of the soil microbial community are directly affected by vegetation and soil properties, because they can provide living environment and nutrient resources for microbial survival [39]. Therefore, it is expected that soil microbial (bacterial and fungal) communities significantly respond to the variations in soil nutrients, salinity, pH, land use, or vegetation [40]. In this study, long-term desertification of grassland resulted in the disappearance of the original vegetation, soil degradation, and the formation of shifting sand dunes. The great change in the soil environment, especially massive losses of soil C and N resources, was unfavorable for the growth and propagation of most soil fungal species, thereby reducing fungal diversity. The observed OTUs and alpha indices including the Shannon–Wiener, Chao, and ACE measures in the SSD were much lower than those in the NCF, showing the significant effect of desertification on the soil fungal community. Conversely, revegetation by planting a CM plantation on a shifting sand dune facilitated the restoration of the soil fungal community due to the improvement in soil health. The diversity of soil fungi increased with the CM plantation age and can be restored to its original state after more than 30 years of coupled succession with the plantation (Table 3). Soil fungal diversity is a useful indicator evaluating the stability and function of an ecosystem [41,42]. It is well known that soil fungi play important roles in the decomposition of SOM. Some studies reported that high amounts of soil mycorrhizal fungi can decrease N loss from soil denitrification and increase the potential of carbon sequestration [43,44].

Ascomycota, Basidiomycota, and Zoopagomycota were considered dominant soil fungal phyla and were detected in all samples. Ullah et al. reported that the three phyla also dominated the soil fungal communities in fluvo-aquic soil and black soil in the Northeast China and North China Plains, and their relative abundance varied with the fertilization regime [42]. Delgado et al. investigated the soil fungal diversity using a high-throughput DNA sequencing method in a low montane humid forest of Ecuador and found Ascomycota and Basidiomycota were the dominant taxa among soil fungal communities [45]. However, the relative abundance of these dominant fungal phyla was different from soil types and showed that geographic separation significantly affected fungal communities, because the compositions in genera and species of fungal communities were different due to the great distinctions in climate, environmental variables, and especially soil nutrient status [20,46,47]. In this study, the alpha diversity, observed species, and the relative abundance of Basidiomycota and Zoopagomycota linearly increased with the plantation age; however, the relative abundance of Ascomycota almost remained unchanged among the SSD, CM plantation, and NCF sites. This phenomenon suggests that many populations of Ascomycota unrelated to edaphic conditions exist in soil, acting as resilient core members that retain the stability of the fungal community, and the succession of the soil fungal community along the sand-fixation plantation development was mainly expressed as the quantitative dynamics of Basidiomycota and Zoopagomycota. In addition, Mucoromycota was also detected in CM19, CM33, and the NCF. Although their relative abundance was very low (<0.1%), they may play important roles in plant growth and community development, because many of them can form symbionts with plant roots.

The dominant genera were significantly different among the sites and exhibited a waning and waxing tendency. Revegetation of the CM plantation on the moving sand dune can significantly alter the dominant genera of the soil fungal community. The relative abundance of *Gloeotinia*, *Saccharomyces*, *Rhizopus*, *Phialemonium*, *Malassezia*, and *Echinoderma* all significantly decreased after the establishment of the CM plantation; simultaneously, many new soil fungal taxa gradually invaded, indicating the effect of vegetation on the soil fungal community. The generic composition and the structure of the fungal community in the SSD were significantly different from those of the CM plantations and NCF; however, they were similar among vegetation-covered sites, especially between CM33 and NCF. The phenomenon indicated that the soil fungal community can be restored by revegetation of the native shrub plantation, even though the original vegetation and soil has been completely degraded into moving sand dunes. In addition, *Fusarium*, one of the dominant genera in vegetation-covered sites, is a widely distributed taxon and was reported in some previous studies [21,45,48]. Although most OTUs cannot be accurately identified at the species level, some dominant species in the SSD and CM-covered sites were observed (Figure 3). The dynamics of their relative abundance directly affected the structure and succession of the soil fungal community, and their ecological functions on the improvements in soil properties and the nutrient cycle and the interactions with bacteria and plants deserve to be studied further in the future.

### 4.3. Relationship between Soil Fungal Community and Soil Factors

Vegetation types, the preference of habitats, the quality of litter inputs, and soil properties are all key factors affecting the soil fungal community during the restoration of degraded ecosystems [49]. The interactions among plants, soil, and microorganisms can determine the structure of the soil fungal community and further affect the ecological processes and ecosystem functions. Among these, vegetation is the primary factor driving soil fungal community succession, because the dynamic of vegetation can alter the quantity and composition of litter, thereby affecting soil nutrients [50]. Soil can be considered the direct factor shaping the soil microbial community in that it can supply almost all the nutrients necessary for microbial survival. In this study, soil fungal communities were clustered into different groups along the plantation development (Figure 1 and Figure 4), showing the synchronous alternations between the vegetation and soil fungal community. The RDA showed that most assayed soil properties, including soil nutrient content and enzymatic activity, positively facilitated the succession of the fungal community. Soil moisture, organic matter, TN, urease, and protease were the main factors affecting the structure of the soil fungal community. Many studies have confirmed that long-term fertilization, land use change, and vegetation conversion significantly alter the structure of the soil fungal community [20,21,39,42,46,48]. However, Yang et al. reported that plant richness had a positive relationship with plant productivity, but fungal richness did not vary with the productivity according to a large-scale investigation [20]. We also observed that Ascomycota insensitively responded to CM plantation development and did not depend on soil properties. This result was in accordance with the report of Ren et al. [46], which investigated the responses of soil bacterial and fungal communities to afforested lands in the Loess Plateau. These results suggest that the relationship between fungal communities and vegetation is very complex, and there may be different response patterns to vegetation types at different scales.

## 5. Conclusions

In this study, we mainly investigated the response of the diversity and structure of the soil fungal community to the reforestation of CM shrub on a shifting sand dune. We found that the establishment of the CM plantation not only did not deplete limited soil nutrients, but it also significantly ameliorated soil properties, nutrient contents, and enzymatic activities. The structure of the soil fungal community gradually reformed and evolved with the development of the plantation. The diversity of the soil fungal community linearly increased with plantation age. During the succession of the fungal community, the relative abundance of dominant taxa showed a waning and waxing characteristic, and the composition and structure of the soil fungal community in CM33 were very close to that of the NCF. The succession of the soil fungal community was directly driven by soil properties, and soil moisture, SOM, TN, urease, and protease were the main affecting factors. The results revealed that the soil fungal community of desertified grassland (even degraded into moving sand dunes) can be reversibly restored to its original state on a human time scale by the afforestation of native shrub.

## Figures and Tables

**Table 1:** Morphological traits of *Caragana microphylla* plantations.

Site	Average Height (cm)	Crown Diameter (cm × cm)	Shoot Number (N/clump)	Vegetation Coverage (%)	Dominant Herbaceous Plant Species under Crown
SSD	-	-	-	<5%	*Agriophyllum squarrosum*, *Setaria viridis*
CM8	70.3 ± 12.6a	70 × 75	10.5 ± 2.8a	60	*Setaria viridis*, *Corispermum sibiricum*, *Salsola collina*, *Bassia dasyphylla*
CM19	84.4 ± 19.2b	95 × 85	21.4 ± 4.8b	80	*Cynachum sibiricum*, *Chenopodium acuminatum*, *B. dasyphylla*, *Eragrostis poaeoides*
CM33	88.5 ± 23.6b	105 × 88	28.3 ± 5.6b	85	*Pennisetum flaccidum*, *Chenopodium acuminatum*, *Artemisia sieversiana*
NCF	135.8 ± 25.7c	150 × 140	50.5 ± 23.5c	90	*Agropyron cristatum*, *P. flaccidum*, *Cleistogenes squarrosa*, *Lespedeza davurica*

Values (means ± SD) are the averages of 50 measurements; different letters within a column indicate a significant difference (*p* < 0.05). SSD: shifting sand dune (0-yr); CM8, CM19, and CM33: 8-, 19-, and 33-year *C. microphylla* plantation, respectively; NCF: natural *C. microphylla* forest.

**Table 2:** Soil properties in different *Caragana microphylla* plantations.

Item	SSD	CM8	CM19	CM33	NCF	ANOVA in Response to Age		
						*R* ^2^	*F*	*p*
Soil moisture (%)	0.161 ± 0.020a	0.290 ± 0.071ab	0.487 ± 0.083ab	0.681 ± 0.277b	1.135 ± 0.318b	0.807	57.39	<0.001
pH	6.782 ± 0.040a	6.877 ± 0.018ab	6.937 ± 0.066ab	6.973 ± 0.145ab	7.042 ± 0.071b	0.598	19.34	0.001
Electrical conductivity (µs cm^−1^)	29.23 ± 3.990a	48.35 ± 4.753b	57.36 ± 2.900bc	66.34 ± 4.920c	80.94 ± 7.230d	0.894	109.8	<0.001
Organic matter (%)	0.043 ± 0.011a	0.189 ± 0.013b	0.259 ± 0.047b	0.539 ± 0.087c	0.977 ± 0.139d	0.931	174.1	<0.001
Total N (%)	0.009 ± 0.003a	0.053 ± 0.008b	0.061 ± 0.005bc	0.069 ± 0.009c	0.083 ± 0.005d	0.737	36.41	<0.001
Total P (%)	0.032 ± 0.001a	0.048 ± 0.006ab	0.056 ± 0.004bc	0.072 ± 0.006c	0.112 ± 0.021d	0.876	91.79	<0.001
Total K (%)	1.972 ± 0.046a	2.192 ± 0.114b	2.229 ± 0.060b	2.319 ± 0.082b	2.353 ± 0.088b	0.634	22.53	<0.001
NH4-N (mg kg^−1^)	2.007 ± 0.583a	2.758 ± 0.149b	3.343 ± 0.259bc	3.785 ± 0.117c	4.198 ± 0.557c	0.795	50.39	<0.001
Available P (mg kg^−1^)	4.700 ± 0.633a	5.654 ± 0.183a	6.731 ± 0.587b	6.981 ± 0.322b	8.423 ± 0.930c	0.819	64.44	<0.001
Available K (mg kg^−1^)	415.2 ± 10.48a	437.5 ± 4.553b	448.4 ± 1.691b	461.5 ± 3.549d	471.6 ± 4.766d	0.868	85.78	<0.001
Urease (mg 100 g^−1^ 24 h^−1^)	0.590 ± 0.115a	3.510 ± 0.710a	8.619 ± 1.592b	17.94 ± 2.346c	32.92 ± 3.916d	0.953	263.1	<0.001
APA (mg g^−1^ h^−1^)	2.485 ± 0.948a	18.87 ± 3.915a	47.313 ± 10.68b	92.50 ± 8.293c	156.9 ± 25.81d	0.954	267.5	<0.001
Protease (mg Tyr g^−1^ 2 h^−1^)	7.321 ± 1.113a	18.30 ± 6.427a	50.82 ± 8.216b	87.93 ± 11.167c	109.6 ± 13.88d	0.944	217.3	<0.001
Glucosidase (μg g^−1^ h^−1^)	0.153 ± 0.016a	0.469 ± 0.057b	0.579 ± 0.065b	0.947 ± 0.120d	1.334 ± 0.155d	0.950	245.2	<0.001
Dehydrogenase (mg TPF kg^−1^ 24 h^−1)^	24.77 ± 8.124a	47.29 ± 1.942a	64.45 ± 5.051a	129.5 ± 23.52b	174.3 ± 61.62b	0.823	60.30	<0.001
POA (µmol g^−1^ 10 min^−1^)	1.784 ± 0.325a	2.523 ± 0.643a	3.167 ± 0.354ab	4.225 ± 0.709b	4.765 ± 1.302b	0.737	36.42	<0.001

Values are means ± SD. SSD: shifting sand dune (0-yr); CM8, CM19, and CM33: 8-, 19-, and 33-year-old *C. microphylla* plantation, respectively; NCF: natural *C. microphylla* forest. APA: alkaline phosphomonoesterase; POA: polyphenol oxidase. *R*^2^, *F*, and *p* values, from linear regression model are given. Means in row followed by the different letters are significantly different based on Fisher’s LSD test (*p* < 0.05).

**Table 3:** Alpha diversity of fungal communities of different samples.

Index	SSD	CM8	CM19	CM33	NCF	ANOVA in Response to Age		
						*R* ^2^	*F*	*p*
ACE	76.895 ± 18.29a	406.52 ± 26.98b	452.93 ± 44.55b	552.65 ± 62.88c	590.6 ± 41.68c	0.704	30.976	<0.001
Chao	68.83 ± 18.45a	411.8 ± 26.53b	457.75 ± 45.83b	566.8 ± 60.97b	599.1 ± 38.61b	0.703	30.745	<0.001
Shannon–Wiener	4.428 ± 0.182a	5.896 ± 0.078b	5.599 ± 0.735b	6.557 ± 0.404c	6.567 ± 0.388c	0.597	19.267	0.001
Observed OTUs	63.7 ± 20.90a	388.8 ± 16.88b	426.5 ± 40.01b	508.6 ± 56.18c	556.5 ± 37.52c	0.693	29.364	<0.001

Values are means ± SD. SSD: shifting sand dune (0-yr); CM8, CM19, and CM33: 8-, 19-, and 33-year-old *C. microphylla* plantation, respectively; NCF: natural *C. microphylla* forest. ACE: abundance-based coverage estimator; Chao: Chao’s species richness estimator. *R*^2^, *F*, and *p* values from linearity regression model are given. Means in row followed by the different letters are significantly different based on Fisher’s LSD test (*p* < 0.05).

## Data Availability

The original contributions presented in the study are included in the article/Supplementary Materials, further inquiries can be directed to the corresponding author.

## Supplementary Materials

The following supporting information can be downloaded at: https://www.mdpi.com/article/10.3390/microorganisms12081545/s1, Figure S1: Observed OTUs rarefaction curves; File S1: Supplementary materials and methods.

## References

[1] Treseder K.K., Lennon J.T. (2015). Fungal traits that drive ecosystem dynamics on land. Microbiol. Mol. Biol. Rev..

[2] Frac M., Hannula S.E., Belka M., Jedryczka M. (2018). Fungal biodiversity and their role in soil health. Front. Microbiol..

[3] Begum N., Qin C., Ahanger M.A., Raza S., Khan M.I., Ashraf M., Ahmed N., Zhang L.X. (2019). Role of arbuscular mycorrhizal fungi in plant growth regulation: Implications in abiotic stress tolerance. Front. Plant Sci..

[4] Almeida B.K., Ross M.S., Stoffella S.L., Sah J.P., Cline E., Sklar F., Afkhami M.E. (2020). Diversity and structure of soil fungal communities across experimental Everglades Tree Islands. Diversity.

[5] Barea J.M., Palenzuela J., Cornejo P., Sanchez-Castro I., Navarro-Fernandez C., Lopez-Garcia A., Estrada B., Azcon R., Ferrol N., Azcon-Aguilar C. (2011). Ecological and functional roles of mycorrhizas in semi-arid ecosystems of Southeast Spain. J. Arid Environ..

[6] Avis P.G., Gaswick W.C., Tonkovich G.S., Leacock P.R. (2017). Monitoring fungi in ecological restorations of coastal Indiana, USA. Restor. Ecol..

[7] van der Heijden M.G.A., Klironomos J.N., Ursic M., Moutoglis P., Streitwolf-Engel R., Boller T., Wiemken A., Sanders I.R. (1998). Mycorrhizal fungal diversity determines plant biodiversity, ecosystem variability and productivity. Nature.

[8] Maltz M.R., Treseder K.K. (2015). Sources of inocula influence mycorrhizal colonization of plants in restoration projects: A meta-analysis. Restor. Ecol..

[9] Emam T. (2016). Local soil, but not commercial AMF inoculum, increases native and non-native grass growth at a mine restoration site. Restor. Ecol..

[10] Wubs E.R.J., van der Putten W.H., Bosch M., Bezemer T.M. (2016). Soil inoculation steers restoration of terrestrial ecosystems. Nat. Plants.

[11] Wardle D.A., Lindahl B.D. (2014). Disentangling global soil fungal diversity. Science.

[12] Monkai J., Hyde K.D., Xu J.C., Mortimer P.E. (2017). Diversity and ecology of soil fungal communities in rubber plantations. Fungal Biol. Rev..

[13] Wang T., Zhu Z.D. (2003). Study on sandy desertification in China-definition of sandy desertification and its connotation. J. Desert Res..

[14] Liu X.M., Zhao H.L. (1993). Comprehensive Strategy for Eco-Environmental Control in Horqin Sand Land.

[15] Su Y.Z., Zhao H.F. (2003). Soil properties and plant species in an age sequence of *Caragana microphylla* plantations in the Horqin Sandy Land, north China. Ecol. Eng..

[16] Wang T., Xue X., Zhou L., Guo J. (2015). Combating aeolian desertification in northern China. Land Degrad. Dev..

[17] Li S.G., Harazono Y., Zhao H.L., He Z.Y., Chang X.L., Zhao X.Y., Zhang T.H., Oikawa T. (2002). Micrometeorological changes following establishment of artificially established *Artemisia* vegetation on desertified sandy land in the Horqin sandy land, China and their implication on regional environmental change. J. Arid. Environ..

[18] Su Y.Z., Zhang T.H., Li Y.L., Wang F. (2005). Changes in soil properties after establishment of Artemisia halodendron and *Caragana microphylla* on shifting sand dunes in semi-arid Horqin Sandy Land, Northern China. Environ. Manag..

[19] Miao R.H., Jiang D.M., Musa A., Zhou Q.L., Guo M.X., Wang Y.C. (2015). Effectiveness of shrub planting and grazing exclusion on degraded sandy grassland restoration in Horqin sandy land in Inner Mongolia. Ecol. Eng..

[20] Yang T., Adams J.M., Shi Y., He J.S., Jing X., Chen L.T., Tedersoo L., Chu H.Y. (2017). Soil fungal diversity in natural grasslands of the Tibetan Plateau: Associations with plant diversity and productivity. New Phytol..

[21] Yang Y., Dou Y.X., Huang Y.M., An S.S. (2017). Links between soil fungal diversity and plant and soil properties on the Loess Plateau. Front. Microbiol..

[22] Liu J.J., Sui Y.Y., Yu Z.H., Shi Y., Chu H.Y., Jin J., Liu X.B., Wang G.H. (2015). Soil carbon content drives the biogeographical distribution of fungal communities in the black soil zone of northeast China. Soil Biol. Biochem..

[23] Li P., Li Y.C., Zheng X.Q., Ding L.N., Ming F., Pan A.H., Lv W.G., Tang X.M. (2018). Rice straw decomposition affects diversity and dynamics of soil fungal community, but not bacteria. J. Soils Sediments.

[24] Zhou J., Jiang X., Zhou B.K., Zhao B.S., Ma M.C., Guan D.W., Li J., Chen S.F., Cao F.M., Shen D.L. (2016). Thirty four years of nitrogen fertilization decreases fungal diversity and alters fungal community composition in black soil in northeast China. Soil Biol. Biochem..

[25] Li H.L., Zhang Y., Wang T.T., Feng S.W., Ren Q., Cui Z.B., Cao C.Y. (2019). Responses of soil denitrifying bacterial communities carrying *nirS*, *nirK*, and *nosZ* genes to revegetation of moving sand dunes. Ecol. Indic..

[26] Wang F., Zhang Y., Xia Y., Cui Z.B., Cao C.Y. (2021). Soil microbial community succession based on *phoD* and *gcd* genes along a chronosequence of sand-fixation forest. Forests.

[27] FAO, FAO/IUSS Working Group WRB (2006). World Reference Base for Soil Resources 2006.

[28] Lin D.Y. (2004). Guidance of Soil Science Experiment.

[29] Kandeler E., Gerber H. (1988). Short-term assay of soil urease activity using colorimetric determination of ammonium. Biol. Fertil. Soils.

[30] Ladd J.N., Butler J.H.A. (1972). Short-term assays of soil proteolytic enzyme activities using proteins and dipeptide derivatives as substrates. Soil Biol. Biochem..

[31] Xu G.H., Zheng H.Y. (1986). Manual of Analytical Methods of Soil Microorganism.

[32] Tabatabai M.A., Page A.L., Millar E.M., Keeney D.R. (1982). Soil enzymes. Methods of Soil Analysis.

[33] Sardans J., Peñuelas J. (2005). Drought decreases soil enzyme activity in a Mediterranean *Quercus ilex* L. forest. Soil Biol. Biochem..

[34] Perucci P., Casucc C., Dumontet S. (2000). An improved method to evaluate the o-diphenol oxidase activity of soil. Soil Biol. Biochem..

[35] Institute of Soil Science, Chinese Academy of Sciences (ISSCAS) (1985). Methods on Soil Microorganism Study.

[36] Kirk P.M., Cannon P.F., Minter D.W., Stalpers J.A. (2008). Ainsworth & Bisby’s Dictionary of the Fungi.

[37] Badiane N.N.Y., Chotte J.L., Pate E., Masse D., Rouland C. (2001). Use of soil enzyme activities to monitor soil quality in natural and improved fallows in semi-arid tropical regions. Appl. Soil Ecol..

[38] Yang H., Guo Z.L., Chu X.L., Man R.Z., Chen J.X., Liu C.J., Tao J., Jiang Y. (2019). Comment on impacts of species richness on productivity in a large-scale subtropical forest experiment. Science.

[39] Ngugi M.R., Fechner N., Neldner V.J., Dennis P.G. (2020). Successional dynamics of soil fungal diversity along a restoration chronosequence post-coal mining. Retor. Ecol..

[40] Suleiman A.K.A., Manoeli L., Boldo J.T., Pereira M.G., Roesch L.F.W. (2013). Shifts in soil bacterial community after eight years of land-use change. Syst. Appl. Microbiol..

[41] Chaer G., Fernandes M., Myrold D., Bottomley P. (2009). Comparative resistance and resilience of soil microbial communities and enzyme activities in adjacent native forest and agricultural soils. Microb. Ecol..

[42] Ullah S., Ai C., Ding W.C., Jiang R., Zhao S.C., Zhang J.J., Zhou W., Hou Y.P., He P. (2019). The response of soil fungal diversity and community composition to long-term fertilization. Appl. Soil Ecol..

[43] Six J., Frey S., Thiet R., Batten K. (2006). Bacterial and fungal contributions to carbon sequestration in agroecosystems. Soil Sci. Soc. Am. J..

[44] Bender S.F., Plantenga F., Neftel A., Jocher M., Oberholzer H.R., Kohl L., Giles M., Daniell T.J., van der Heijden M.G.A. (2014). Symbiotic relationships between soil fungi and plants reduce N_2_O emissions from soil. ISME J..

[45] Delgado E.F., Valdez A.T., Covarrubias S.A., Tosi S., Nicola L. (2022). Soil fungal diversity of the Aguarongo Andean forest (Ecuador). Biology.

[46] Ren C.J., Liu W.C., Zhao F.Z., Zhong Z.K., Deng J., Han X.H., Yang G.H., Feng Y.Z., Ren G.X. (2019). Soil bacterial and fungal diversity and compositions respond differently to forest development. Catena.

[47] Fu Z.Q., Chen Q., Lei P.F., Xiang W.H., Ouyang S., Chen L. (2021). Soil fungal communities and enzyme activities along local tree species diversity gradient in subtropical evergreen forest. Forests.

[48] Hu X., Liu J., Wei D., Zhu P., Cui X., Zhou B., Chen X., Jin J., Liu X., Wang G. (2017). Effects of over 30-year of different fertilization regimes on fungal community compositions in the black soils of Northeast China. Agric. Ecosyst. Environ..

[49] Abu Hanif M., Guo Z.M., Moniruzzaman M., He D., Yu Q.S., Rao X.Q., Liu S.P., Tan X.P., Shen W.J. (2019). Plant taxonomic diversity better explains soil fungal and bacterial diversity than functional diversity in restored forest ecosystems. Plant.

[50] Miki T., Ushio M., Fukui S., Kondoh M. (2010). Functional diversity of microbial decomposers facilitates plant coexistence in a plant-microbe-soil feedback model. Proc. Natl. Acad. Sci. USA.

