# Supplementary material for: Response of Soil Fungal Community to Reforestation on Shifting Sand Dune in the Horqin Sandy Land, Northeast China
Source: Microorganisms. 2024 Jul 28;12(8):1545. doi: 10.3390/microorganisms12081545 (PMC11356087; doi:10.3390/microorganisms12081545)
Supplement: Supplementary file 1 [file microorganisms-12-01545-s001.zip › File S1.pdf]

Supplementary Materials and Methods

Soil enzymatic activity determination

Soil urease activity was determined using urea as the substrate, and the released ammonium was assayed colorimetrically at 460 nm [1]. Protease activity was determined using the method of Ladd and Butler [2]. 1 g of the soil samples was incubated for 2 h in 5 mL of a buffered casein solution (pH 8.1) and 5 mL of TRIS buffer (50 mM, pH 8.1) at 50 °C. The released aromatic amino acids were extracted with trichloroacetic acid (0.92 M) and measured colorimetrically by using Folin-Ciocalteu reagent. The activity was expressed as mg Tyr (tyrosine equivalents) kg^{-1} soil h^{-1}. Glucosidase activity was determined using the method described by Xu and Zheng [3]. The activity of soil alkaline phosphomonoesterase was measured using the original method of Tabatabai [4], with some modifications by Sardans and Peñuelas [5]. The *p*-nitrophenol content was calculated by referring to a calibration curve obtained with standards containing 0, 10, 20, 30, 40, and 50 mg L^{-1} of *p*-nitrophenol, and the activity was expressed as mg *p*-nitrophenol (pNP) per kilogram dry matter and incubation time. Dehydrogenase was measured following the method described in ISSCAS [6]. Briefly, 5 g soil was incubated in 5 mL 5 g L^{-1} triphenyltetrazolium chloride (TTC) and 2 mL 0.1 M glucose at 37 °C for 12 h. The reactions were terminated by 0.25 mL 98% H_2SO_4, and the products were extracted for 30 min with 5 mL toluene on a shaker. After centrifugation, the triphenyl formazone (TPF) dissolved in toluene was assayed at 492 nm. The activity was expressed as mg of TPF released kg^{-1} soil h^{-1}. Polyphenol oxidase activity was measured with the method described by Perucci et al. [7], and expressed as mmoles of catechol oxidised kg^{-1} soil h^{-1}.

DNA extraction, ITS rDNA sequencing, and data processing and analysis

Microbial DNA was extracted from 0.3 g fresh soil sample using the soil DNA extraction kit (Sangon Biotech, Shanghai, China) following the manufacturer's instructions. Next generation sequencing library preparations and Illumina MiSeq sequencing were conducted at GENEWIZ, Inc. (Suzhou, China). DNA samples were quantified using a Qubit 2.0 Fluorometer (Invitrogen, Carlsbad, CA, USA). 50-100 ng DNA was used to generate amplicons using a panel of primers designed by GENEWIZ (GENEWIZ, Inc., South Plainfield, NJ, USA). Oligonucleotide primers were designed to anneal to the relatively conserved sequences spanning fungi ITS regions. ITS2 region was amplified using forward primer containing sequence "GTGAATCATCGARTC" and reverse primer containing sequence "TCCTCCGCTTATTGAT". Besides the

ITS target-specific sequences, the primers also contain adaptor sequences allowing uniform amplification of the library with high complexity ready for downstream NGS sequencing on Illumina Miseq platform. DNA libraries were validated by Agilent 2100 Bioanalyzer (Agilent Technologies, Palo Alto, CA, USA), and quantified by Qubit 2.0 Fluorometer. DNA libraries were multiplexed and loaded on an Illumina MiSeq instrument according to manufacturer's instructions (Illumina, San Diego, CA, USA). Sequencing was performed using a 2x300/250 paired-end (PE) configuration; image analysis and base calling were conducted by the MiSeq Control Software (MCS) embedded in the MiSeq instrument.

The QIIME data analysis package was used for ITS rDNA data analysis. The forward and reverse reads were joined and assigned to samples based on barcode and truncated by cutting off the barcode and primer sequence. Quality filtering on joined sequences was performed and sequence which did not fulfill the following criteria were discarded: sequence length < 200 bp, no ambiguous bases, mean quality score $\geqslant$ 20. Then the sequences were compared with the reference database (RDP Gold database) using UCHIME algorithm to detect chimeric sequence, and then the chimeric sequences were removed. The effective sequences were used in the final analysis. Sequences were grouped into operational taxonomic units (OTUs) using the clustering program VSEARCH (1.9.6) against the UNITE ITS database (https://unite.ut.ee/) at 97% cutoff. The Ribosomal Database Program (RDP) classifier was used to assign taxonomic category to all OTUs at confidence threshold of 0.8. The RDP classifier uses the UNITE ITS database which has taxonomic categories predicted to the species level. Sequences were rarefied prior to calculation of alpha and beta diversity statistics. Alpha diversity indexes including the Shannon-Wiener index (SW), Chao's species richness estimator (Chao), and abundance-based coverage estimator (ACE) were calculated in QIIME from rarefied samples. Hierarchical clustering analysis was performed and Unweighted Pair Group Method with arithmetic means (UPGMA) tree was built to differentiate the fungal community structures of different sites.

References

[1] Kandeler, E.; Gerber, H. Short-term assay of soil urease activity using colorimetric determination of ammonium. *Biol. Fertil. Soils* **1988**, *6*, 68–72. https://doi.org/10.1007/BF00257924.

[2] Ladd, J.N.; Butler, J.H.A. Short-term assays of soil proteolytic enzyme activities using proteins and dipeptide derivatives as substrates. *Soil Biol. Biochem.* **1972**, *4*, 19–30. https://doi.org/10.1016/ 0038-0717(72)90038-7.

[3] Xu, G.H.; Zheng, H.Y. *Manual of analytical methods of soil microorganism*. China Agriculture Press. Beijing, **1986**, pp 266–269.

[4] Tabatabai, M.A. Soil enzymes. In: Page, A.L., Millar, E.M., Keeney, D.R. (Eds.). *Methods of Soil Analysis*. ASA and SSSA, Madison, WI, *1982*; pp. 501–538.

[5] Sardans, J.; Peñuelas, J. Drought decreases soil enzyme activity in a Mediterranean Quercus ilex L. forest. *Soil Biol. Biochem.* **2005**, *37*, 455–461. https://doi.org/10.1016/j.soilbio.2004.08.004.

[6] Institute of Soil Science, Chinese Academy of Sciences (ISSCAS). *Methods on Soil Microorganism Study*. Science Press: Beijing, **1985**; pp. 260–275.

[7] Perucci, P.; Casucc, C.; Dumontet, S. An improved method to evaluate the o-diphenol oxidase activity of soil. *Soil Biol. Biochem.* **2000**, *32*, 1927–1933. https://doi.org/10.1016/S0038-0717(00)00168-1.
