# Supplementary figures and images for: Response of Soil Fungal Community to Reforestation on Shifting Sand Dune in the Horqin Sandy Land, Northeast China
Source: Microorganisms. 2024 Jul 28;12(8):1545. doi: 10.3390/microorganisms12081545 (PMC11356087; doi:10.3390/microorganisms12081545)

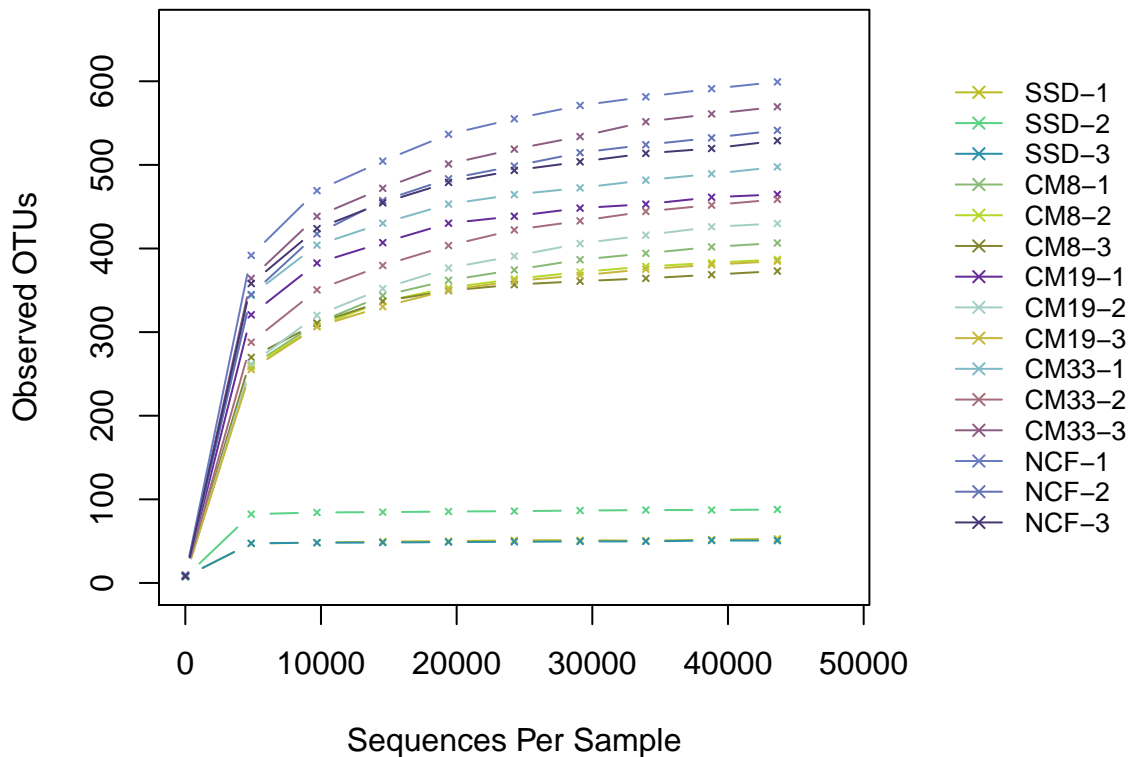

Supplement: Supplementary file 1 [file microorganisms-12-01545-s001.zip › Figure S1.pdf]
